# The path linking disease severity and cognitive function with quality of life in Parkinson’s disease: the mediating effect of activities of daily living and depression

**DOI:** 10.1186/s12955-021-01740-w

**Published:** 2021-03-17

**Authors:** Yao He, Yuling Tian, Hongjuan Han, Jing Cui, Xiaoyan Ge, Yao Qin, Yanhong Luo, Wenlin Bai, Hongmei Yu

**Affiliations:** 1 Shanxi Provincial Key Laboratory of Major Diseases Risk Assessment, Department of Health Statistics, School of Public Health, Shanxi Medical University, 56 South XinJian Road, Taiyuan, 030001 People’s Republic of China; 2 Department of Neurology, First Hospital of Shanxi Medical University, Taiyuan, People’s Republic of China

**Keywords:** Parkinson’s disease, Multiple mediation model, Activities of daily living, Depression, Quality of life

## Abstract

**Background:**

Research on quality of life (QOL) with Parkinson’s disease (PD) has examined direct influencing factors, not mediators. The study aim was to explore whether PD severity and poor cognitive function may decrease physical and mental QOL by reducing activities of daily living (ADL) and increasing depression in sequence.

**Methods:**

We conducted a cross-sectional questionnaire study of 150 PD hospital patients in China. PD severity, cognitive function, ADL, depression, and QOL were evaluated. We used structural equation modeling to analyze the mediating effects of ADL and depression on the association between PD severity/cognition and the physical health and mental health component summary scores measured by the SF36 quality of life instrument.

**Results:**

There was a significant mediating effect of PD severity on physical health via ADL and depression (95% CI: − 0.669, − 0.026), and a significant direct effect (*p* < 0.001). The mediating effect of PD severity on mental health via ADL and depression was significant (95% CI: − 2.135, − 0.726), but there was no direct effect (*p* = 0.548). There was a significant mediating effect of cognitive function on physical health via ADL and depression (95% CI: 0.025, 0.219) and a significant direct effect (*p* < 0.001). The mediating effect of cognitive function on mental health via ADL and depression was significant (95% CI: 0.256, 0.645), but there was no direct effect (*p* = 0.313). The physical health models showed a partial mediation, and the mental health models showed a complete mediation, of ADL and depression.

**Conclusions:**

PD severity and cognitive function increase depression by reducing ADL, leading to lower QOL, and directly or indirectly affect physical health and mental health through different pathways.

## Introduction

Parkinson’s disease (PD) is a chronic progressive neurodegenerative disorder that affects middle-aged and older people [1]. Non-motor PD symptoms, such as cognitive impairment and depression, have attracted increasing attention and may exacerbate disability, affect quality of life (QOL), and shorten life expectancy [2]. The American economist Galbraith first proposed the concept of QOL in 1958 and it has been widely used as a sociological indicator [3]. QOL is usually classified into two aspects which reflect physiological and psychological QOL, respectively [4].

Common neurological complications of PD include cognitive impairment and depression; stage 4 or higher on the Hoehn and Yahr (HY) Scale indicates greater cognitive impairment [5]. As the disease progresses, the incidence of cognitive impairment gradually increases and eventually develops into Parkinsonism dementia [6]. The pathogenesis of PD and its related complications, such as depression and cognitive impairment, currently remain unclear.

The assessment of activities of daily living (ADL) is an important indicator of older people’s ability to take care of themselves [7]. Chronic diseases are important influencing factors of physical health in older people. Older people who have multiple chronic diseases gradually experience impaired physical motor function and reduced ADL [8]. Depression is a common psychological disorder and is often more prevalent in patients than in health people [9]. Research indicates that PD severity is positively correlated with depression accompanied by fatigue, sleep disturbance, appetite loss, guilt, inattention, and other symptoms [10]. Older people with poor daily self-care ability often experience a heavy psychological burden, which strongly affects ADL and increases the risk of death. There is evidence that lower ADL leads to greater depression [11]. ADL and depression are mediators in some chronic diseases [12]. A study by Chen et al. [13] demonstrated an indirect effect of cognitive impairment and pain on psychological and physiological changes through ADL and depression. This indicates that PD severity and cognitive function are likely to indirectly affect QOL via a decline in ADL and an increase in depression.

In recent years, the QOL of patients with chronic diseases such as PD, cognitive impairment and depression has received widespread attention. The number and severity of chronic diseases are inversely related to QOL, especially physical health [14]. Moreover, the physical and psychological barriers caused by chronic conditions have different effects on physical and mental health [15]. PD severity and cognitive impairment have a negative impact on QOL, but their likely pathways of influence (and their effect on aspects of QOL) may differ. Therefore, we proposed the following hypotheses: (1) ADL and depression are two chain mediators on the path linking PD severity and cognitive function with QOL; (2) There are differences in the mechanisms of influence of PD severity and cognitive function on physical and mental health.

## Materials and methods

### Participants

Between March 2017 and March 2018, 150 outpatients and inpatients from the Department of Neurology in the First Hospital of Shanxi Medical University in China were recruited to this cross-sectional study. Our study received ethics approval from Shanxi Medical University Ethics Committee and informed consent was obtained from all participants. The selection criteria for patients were as follows: (1) Diagnosis of PD in accordance with the 2015 PD criteria of the International Association for Dyskinesia. (2) Ability to understand and complete all questionnaires. The exclusion criteria were: (1) Parkinson’s syndrome caused by other factors, like brain trauma. (2) Patients with idiopathic tremor. We used face-to-face interviews to collect data, and investigators monitored and assisted participants in completing the questionnaires. Patients with PD were asked to provide general information about gender, age, marital status, educational level, family population, smoking and drinking behavior.

### Patient assessment

The Modified Hoehn and Yahr (HY) Scale [16] was used to assess PD severity. Compared with the original HY scale, the Modified HY scale contains 0.5 level increments. Advantages of the HY scale include its wide utilization and suitability for assessing PD severity. It provides an overall assessment of severity based on functional disability. Higher HY scores indicate worse disease state [17].

The Mini-Mental State Examination (MMSE) [18] was used to assess cognitive function, which scores range from 0 to 30. A score of 23 points or less is generally considered to indicate cognitive impairment. The MMSE has satisfactory predictive validity and is an adequate measure of cognition.

The Unified Parkinson’s Disease Rating Scale (UPDRS II) [19] was used to evaluate ADL. The UPDRS has been used in studies of early PD, mild PD, moderate but stable PD, and motion fluctuations, and is increasingly used as a gold standard reference scale. The UPDRS II contains 13 items relating to symptom interference with ADL. Scores range from 0 to 52, higher scores indicate lower ADL [20].

The Geriatric Depression Scale (GDS) [21] was used to assess depression over the last week. The GDS is a self-administered, 30-item yes/no questionnaire. The GDS performs well in differentiating depressed from non-depressed PD patients and is a reliable and valid self-rating depression screening scale for older people [22].

The MOS 36-item Short-Form Health Survey (SF-36) [23] was used to measure QOL. The SF-36 is a well-validated 36-item general health status measure and provides two summary scores: physical component summary (PCS) and mental component summary (MCS). Higher SF-36 scores indicate better QOL [24].

### Statistical analysis

We used EpiData 3.1 (EpiData Association, Odense, Denmark) to record data twice to ensure data accuracy and reliability. We used SAS 9.4 (SAS Institute, Cary, NC, USA) for statistical description, *t*-tests or one-way analysis of variance to describe demographic characteristics, and examined the difference between MCS and PCS scores. Spearman’s rank correlation coefficients were used to explore the associations among the factors affecting QOL. A *p* value of 0.05 was considered significant.

Multiple mediation models were developed with PD severity and cognitive function as the independent variables, PCS and MCS as the dependent variables, and ADL and depression as chain mediators [24]. We used a maximum likelihood structural equation model (ML-SEM) for variable analysis, as this type of model has robust test efficiency when the sample data set is small and subject to skewed distribution, especially for complex models [25]. Mediation analysis was performed using Amos 20.0 (IBM SPSS, Armonk NY). We used the Monte Carlo extension method to test the multiple mediation models, as this method is not dependent on a specific method of estimation or software package, and so is very versatile. The confidence interval (CI) was estimated using the Monte Carlo method, if the interval did not contain a zero, the mediation effect was significant [26].

## Results

### Demographic and general characteristics

A total of 150 PD patients participated (mean age ± standard deviation: 66.37 ± 8.73 years; range: 34–84 years). There were no differences in MCS score according to lifestyle factors, but there were significant differences in PCS score according to age, marital status, and educational backgrounds. The latter factors were therefore included as covariates in the mediation analysis (Table 1).

**Table 1:** Demographics and general characteristics by physical and mental quality of life (N = 150)

	n (%)	MCS		PCS	
		Mean (SD)	*p*	Mean (SD)	*p*
Gender					
Male	85 (56.7)	50.5 (12.5)	0.097	31.4 (13.9)	0.253
Female	65 (43.3)	47.0 (13.2)		28.7 (14.4)	
Age (years)					
≤ 40	1 (0.7)	49.9 (0)	0.507	27.1 (0)	0.028
41–59	33 (22.0)	51.3 (14.0)		36.0 (15.0)	
≥ 60	116 (77.3)	48.3 (12.6)		28.6 (13.6)	
Marital status					
Married	137 (91.3)	49.4 (12.8)	0.222	31.1 (14.2)	0.014
Widowed	13 (8.7)	44.8 (13.6)		21.0 (10.7)	
Education					
No formal education	10 (6.7)	47.0 (13.9)	0.131	19.8 (11.5)	0.002
High school and below	119 (79.3)	48.2 (12.3)		29.7 (13.5)	
Junior college and above	21 (14.0)	54.2 (14.8)		37.8 (15.6)	
Family population					
2	6 (4.0)	56.9 (13.9)	0.304	30.40 (11.4)	0.075
3	49 (32.7)	48.6 (12.3)		33.91 (13.3)	
≥ 3	95 (63.3)	48.7 (13.1)		28.25 (14.5)	
Smoking					
Yes	34 (22.7)	47.9 (14.1)	0.595	31.08 (12.9)	0.676
No	116 (77.3)	49.3 (12.6)		29.92 (14.5)	
Drinking					
Yes	30 (20.0)	49.3 (14.2)	0.862	29.32 (12.2)	0.709
No	120 (80.0)	48.9 (12.6)		30.40 (14.6)	

SD: standard deviation, MCS: mental component summary, PCS: physical component summary

The correlations between PD severity, cognitive function, ADL, depression, PCS, and MCS were significant (*p* < 0.01). PD severity and depression were negatively correlated with PCS and MCS (r =  − 0.583 and r =  − 0.420). Cognitive function was positively correlated with PCS and MCS (r = 0.526 and r = 0.435). ADL was negatively correlated with cognitive function, PCS and MCS (r =  − 0.459, r =  − 0.666, and r =  − 0.410) and positively correlated with PD severity and depression (r = 0.463 and r = 0.560) (Table 2).

**Table 2:** Correlations between factors affecting ADL

Variables	H-Y	MMSE	GDS	UPDRS II	MCS	PCS
H-Y	–					
MMSE	− 0.514**	–				
GDS	0.519**	− 0.488**	–			
UPDRS II	0.463**	− 0.459**	0.560**	–		
MCS	− 0.420**	0.435**	− 0.769**	− 0.410**	–	
PCS	− 0.583**	0.526**	− 0.571**	− 0.666**	0.319**	–
Mean ± SD	2.62 ± 1.33	22.75 ± 5.19	12.68 ± 7.58	12.51 ± 7.71	48.96 ± 12.88	30.19 ± 14.15

H-Y: Modified Hoehn and Yahr Scale, MMSE: Mini-Mental State Examination, GDS: Geriatric Depression Scale, UPDRS II: Unified Parkinson’s Disease Rating Scale, MCS: mental component summary, PCS: physical component summary, SD: standard deviation

**p < 0.01

### Mediation analysis

We constructed the multiple mediation model with PD severity or cognitive function as independent variables, ADL and depression as chain mediator variables, PCS and MCS as dependent variables.

### Multiple mediation model of the effect of PD severity on QOL

**Fig. 1:**
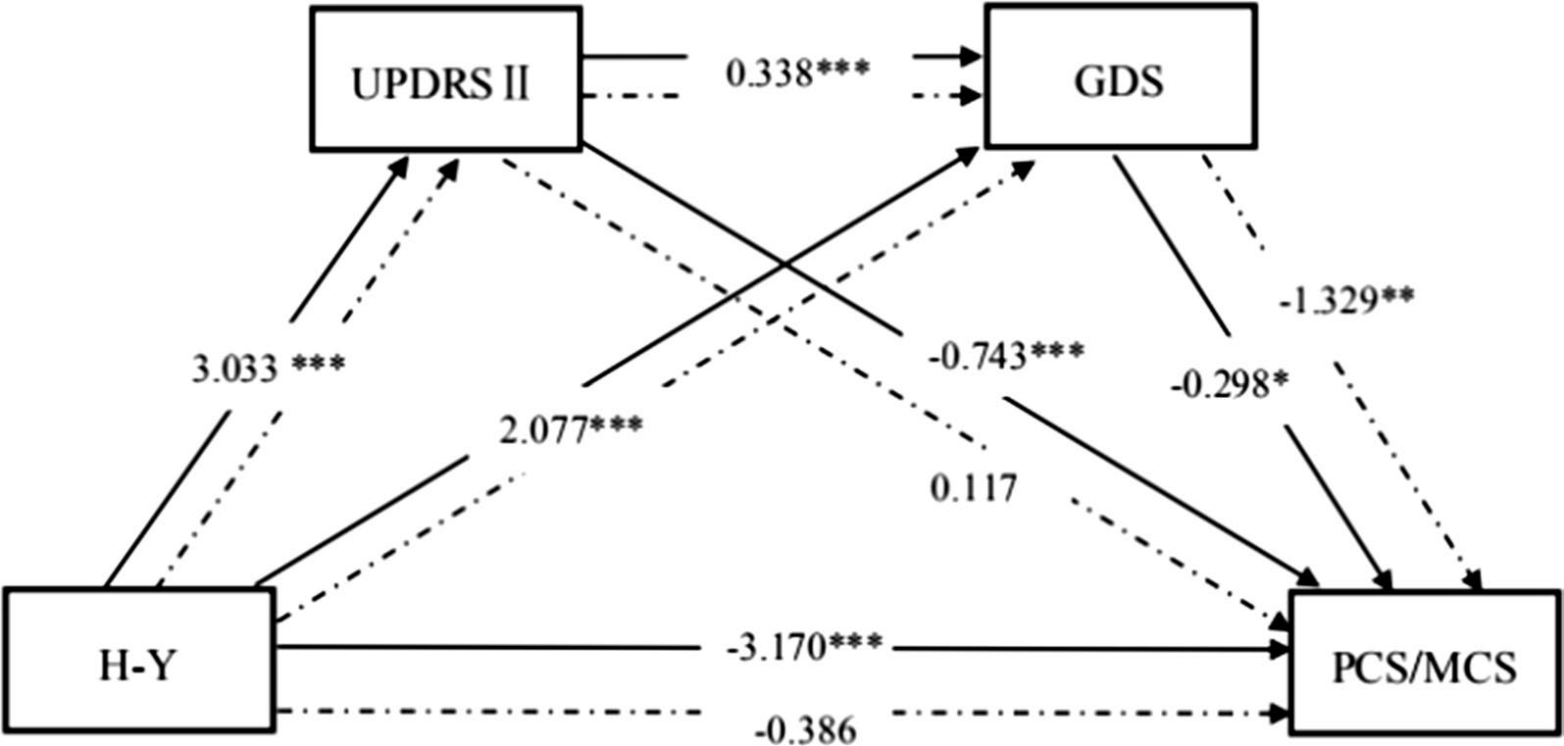
Multiple mediation model with ADL and depression as chaining mediators (PD severity as independent variable). HY: Modified Hoehn and Yahr Scale, UPDRS II: Unified Parkinson’s Disease Rating Scale, GDS: Geriatric Depression Scale, PCS: physical component summary, MCS: mental component summary. The solid lines indicate the PCS model and the dotted lines indicate the MCS model. Control variables: age, marital status, and education level. ***p < 0.001; *p < 0.05

As Fig. 1 and Table 3 show, in the multiple mediation model with PD severity as the independent variable, ADL and depression as the mediators, PCS as the dependent variable, all path coefficients were significant (*p* < 0.05), and the direct effect of PD severity on PCS was significant (path coefficient =  − 3.170, *p* < 0.001), indicating that ADL and depression may be partial mediators that affect PCS.

**Table 3:** Path coefficients for the model showing the effect of PD severity on QOL

Path			Path coefficient	*p*
H-Y	→	UPDRS II	3.033	< 0.001
UPDRS II	→	GDS	0.338	< 0.001
H-Y	→	GDS	2.077	< 0.001
GDS	→	MCS	− 1.329	< 0.001
H-Y	→	MCS	− 0.386	0.548
UPDRS II	→	MCS	0.117	0.302
GDS	→	PCS	− 0.298	0.029
H-Y	→	PCS	− 3.170	< 0.001
UPDRS II	→	PCS	− 0.743	< 0.001

HY: Modified Hoehn and Yahr Scale, UPDRS II: Unified Parkinson’s Disease Rating Scale, GDS: Geriatric Depression Scale, PCS: physical component summary, MCS: mental component summary

In the multiple mediation model with MCS as the dependent variable, there was no relationship between ADL and MCS (path coefficient = 0.117, *p* = 0.302), but other path coefficients were significant (*p* < 0.05). The direct effect of PD severity on MCS was not significant (path coefficient =  − 0.386, *p* = 0.548), indicating that ADL and depression may be complete mediators that affect MCS (Fig. 1 and Table 3).

The path coefficient test results for the effect of PD severity on QOL are shown in Table 3. The mediation effect of the path PD severity → ADL → Depression → PCS was significant (95% CI: − 0.669, − 0.026). The results indicate that PD severity increases depression by reducing ADL, ultimately leading to a decline in PCS. Furthermore, the direct effect of PD severity on PCS was significant (*p* < 0.001). The mediation effect of the path PD severity → ADL → Depression → MCS was significant (95% CI: − 2.135, − 0.726), indicating that PD severity increases depression by reducing ADL, ultimately leading to a decline in MCS. There was no direct effect of PD severity on MCS (*p* = 0.548). Thus, ADL and depression were complete mediators on the path of PD severity effects on mental health, and partial mediators on the path of PD severity effects on physical health.

### Multiple mediation model of the effect of cognitive function on QOL

**Fig. 2:**
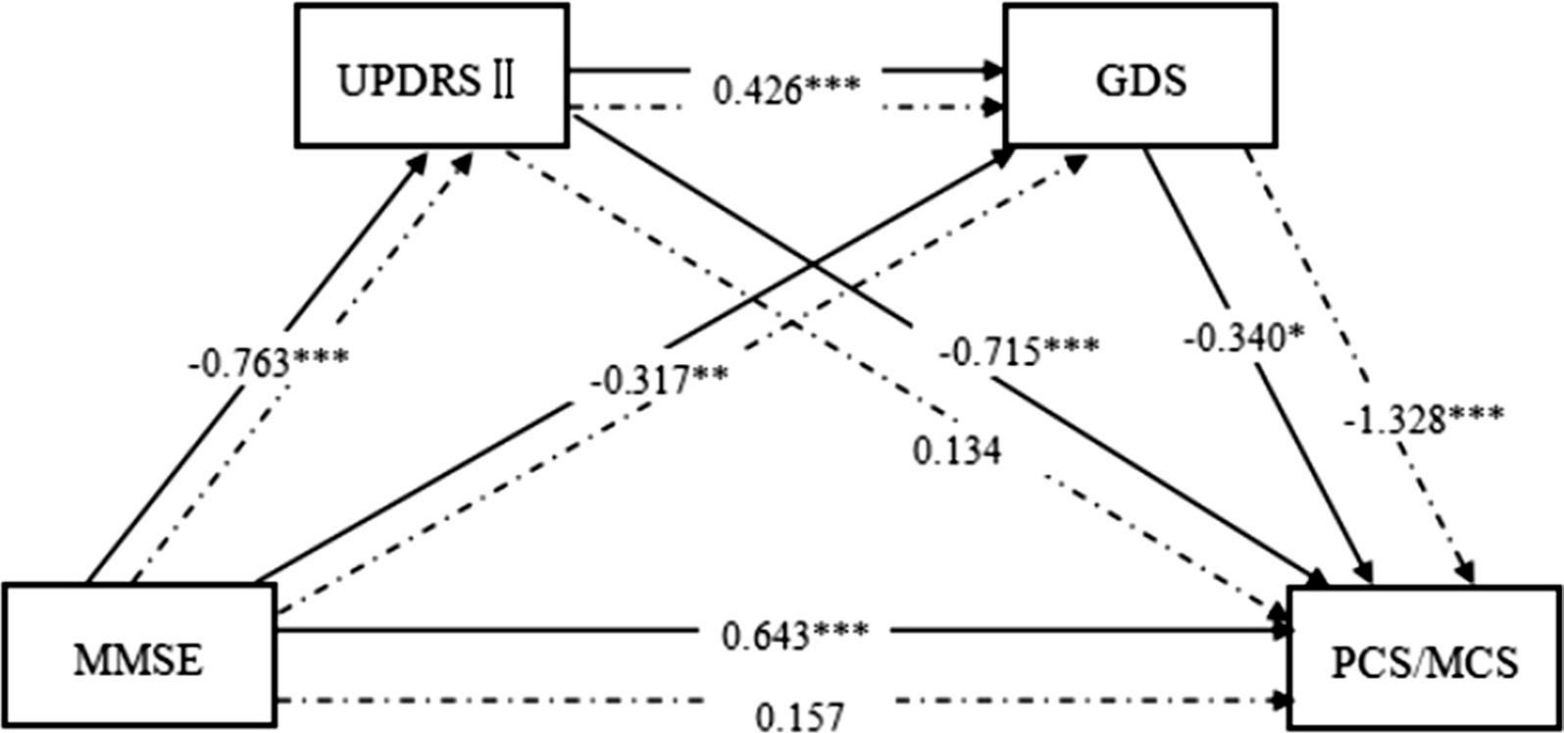
Multiple mediation model with ADL and depression as chaining mediators (cognitive function as independent variable). MMSE: Mini-Mental State Examination, UPDRS II: Unified Parkinson’s Disease Rating Scale, GDS: Geriatric Depression Scale, PCS: physical component summary, MCS: mental component summary. The solid lines indicate the PCS model and the dotted lines indicate the MCS model. Control variables: age, marital status, and education level. ***p < 0.001; *p < 0.05

As Fig. 2 and Table 4 show, in the multiple mediation model with cognitive function as the independent variable, ADL and depression as the mediators, PCS as the dependent variable, all path coefficients were significant (*p* < 0.05) and the direct effect of cognitive function on PCS was significant (path coefficient = 0.643, *p* < 0.001), indicating that ADL and depression may be partial mediators that affect PCS.

**Table 4:** Path coefficients for the model showing the effect of cognitive function on QOL

Path			Path coefficient	*p*
MMSE	→	UPDRS II	− 0.763	< 0.001
UPDRS II	→	GDS	0.426	< 0.001
MMSE	→	GDS	− 0.317	0.004
GDS	→	MCS	− 1.328	< 0.001
MMSE	→	MCS	0.157	0.313
UPDRS II	→	MCS	0.134	0.234
GDS	→	PCS	− 0.340	0.010
MMSE	→	PCS	0.643	< 0.001
UPDRS II	→	PCS	− 0.715	< 0.001

MMSE: Mini-Mental State Examination, UPDRS II: Unified Parkinson’s Disease Rating Scale, GDS: Geriatric Depression Scale, PCS: physical component summary, MCS: mental component summary

In the multiple mediation model with MCS as the dependent variable, there was no relationship between ADL and MCS (path coefficient = 0.134, *p* = 0.234), but the other path coefficients were significant (*p* < 0.05). The direct effect of cognitive function on MCS was not significant (path coefficient = 0.157, *p* = 0.313), indicating that ADL and depression may be complete mediators of MCS (Fig. 2 and Table 4).

The path coefficient test results of the effect of cognitive function on QOL are shown in Table 4. The mediation effect of the path Cognition → ADL → Depression → PCS was significant (95% CI: 0.025, 0.219), which indicates that cognitive function may increase depression by reducing ADL, ultimately leading to a decline in PCS. The mediation effect of the path Cognition → ADL → Depression → MCS was significant (95% CI: 0.256, 0.645), indicating that cognitive function may exacerbate depression by reducing ADL, leading to a decline in MCS. There was no direct effect of cognitive function on MCS (*p* = 0.313), but the direct effect of cognitive function on PCS was significant (*p* < 0.001). Thus, ADL and depression were complete mediators on the path of cognitive function effects on mental health, and partial mediators on the path of cognitive function effects on physical health.

## Discussion

We formulated a multiple mediation model containing ADL and depression as multiple mediators. The PCS models showed a partial mediation effect of ADL and depression, and the MCS models showed a complete mediation effect of ADL and depression. Lower physiological function has been shown to be significantly associated with lower QOL [27]. Motor dysfunction and pain caused by PD lead to reduced ADL, which affects depression, and further affects QOL [28].

Previous studies indicate a negative correlation between ADL and depression in older people [29]. The present findings are consistent with previous results. PD patients with depression have a higher disability rate, and depression accelerates disease progression, reducing treatment compliance and self-care ability [30]. PD can cause physical dysfunction in patients, leading to a decline in ADL, which seriously affects QOL [31]. Our results support these previous findings by showing that a decline in ADL may increase depression, which has a negative impact on QOL. This partly explains the mechanism underlying the effect of ADL on QOL.

In contrast to previous studies, we used PCS and MCS as dependent variables to assess the influence of PD severity and cognitive function on the two dependent variables through two chain mediators: ADL and depression. The results showed that PD severity and cognitive function directly or indirectly affect physical health and mental health through different pathways. Specifically, ADL and depression were only partial mediators in the PCS model, but complete mediators in the MCS model. This indicates that PD severity and cognitive function affect MCS entirely through ADL and depression, and there may be other potential mechanisms in addition to this pathway for PCS.

Mediation models are powerful tools for evaluating causal processes in psychomedicine because they help explain the often complex relationships between the physical and mental areas of human function [32]. Since currently available drugs cannot control disease progression, patient compliance and satisfaction are frustratingly low. In addition, the current treatment of PD is limited to symptom improvement of the difficult symptoms of the disease, and the measurement of QOL has always been emphasized as a result indicator to measure the impact of the disease on the life of patients [33]. Our results suggested that QOL of patients with PD is affected by two chain mediators: ADL and depression. In clinical and preventive health care of PD, the non-motor function and cognitive status should be controlled at the beginning of the chain and targeted prevention and intervention measures should be taken to improve patients' ADL and depression. Therefore, it’s important to raise awareness of the role of non-motor function in the lives of patients with PD, and giving particular attention to the early detection of potential symptoms is important. Heath care providers have to increase the vigilance of initial cognitive function and depressive symptoms to promote early treatment of symptoms, to provide a personalized schedule and structure to help maintain a satisfactory level of activity and enrichment and to facilitate early management of symptoms enhancing the level of intermediary process, so as to improve QOL in patients with PD.

Although this was a detailed study of QOL, there were some study limitations. First, data were collected from only one hospital and a local population was targeted. Therefore, it may be unwise to generalize too much from the present findings. Second, we found that ADL and depression were partial mediators of the association between PD severity and cognitive function and PCS. Although the models developed here seemed robust, additional studies are needed to identify other potential mechanisms.

## Conclusions

The multiple mediation effect model proposed in this study explored the mechanism underlying the effect of PD severity and cognitive function on QOL, and further revealed the different mechanisms for physical and mental QOL. ADL and depression are two mediators in the path linking PD severity and cognitive function with QOL. The findings suggest that the QOL of PD patients could be improved by increasing their ADL and alleviating depressive symptoms.

## Data Availability

The datasets used and/or analysed during the current study are available from the corresponding author on reasonable request.

## Abbreviations

QOL: Quality of life
PD: Parkinson’s disease
ADL: Activities of daily living
PCS: Physical component summary
MCS: Mental component summary
CI: Confidence interval
HY: The Modified Hoehn and Yahr Scale
MMSE: The Mini-Mental State Examination
UPDRS II: The Unified Parkinson’s Disease Rating Scale
GDS: The Geriatric Depression Scale
SF-36: The MOS 36-item Short-Form Health Survey
ML-SEM: Maximum likelihood structural equation model
SD: Standard deviation
